# A systematic review of hand-hygiene and environmental-disinfection interventions in settings with children

**DOI:** 10.1186/s12889-020-8301-0

**Published:** 2020-02-06

**Authors:** Leanne J. Staniford, Kelly A. Schmidtke

**Affiliations:** Department of Psychology, Manchester Metropolitan University, Brooks Building, 53 Bonsall Street, Manchester, M15 6GX England

**Keywords:** Hand-hygiene, Handwashing, Disinfection, Health behavior, Public health

## Abstract

**Background:**

Helping adults and children develop better hygiene habits is an important public health focus. As infection causing bacteria can live on one’s body and in the surrounding environment, more effective interventions should simultaneously encourage personal-hygiene (e.g. hand-hygiene) and environmental-disinfecting (e.g. cleaning surfaces). To inform the development of a future multi-faceted intervention to improve public health, a systematic literature review was conducted on behavior change interventions designed to increase hand-hygiene and environmental-disinfecting in settings likely to include children.

**Methods:**

The search was conducted over two comprehensive data-bases, Ebsco Medline and Web of Science, to locate intervention studies that aimed to increase hand-hygiene or environmental-disinfecting behavior in settings likely to include children. Located article titles and abstracts were independently assessed, and the full-texts of agreed articles were collaboratively assessed for inclusion. Of the 2893 titles assessed, 29 met the eligibility criteria. The extracted data describe the Behavior Change Techniques (version 1) that the interventions employed and the interventions’ effectiveness. The techniques were then linked to their associated theoretical domains and to their capability-opportunity-motivation (i.e., COM-B model) components, as described in the Behavior Change Wheel. Due to the heterogeneity of the studies’ methods and measures, a meta-analysis was not conducted.

**Results:**

A total of 29 studies met the inclusion criteria. The majority of interventions were designed to increase hand-hygiene alone (*N* = 27), and the remaining two interventions were designed to increase both hand-hygiene and environmental-disinfecting. The most used techniques involved shaping knowledge (*N* = 22) and antecedents (*N* = 21). Interventions that included techniques targeting four or more theoretical domains and all the capability-opportunity-motivation components were descriptively more effective.

**Conclusions:**

In alignment with previous findings, the current review encourages future interventions to target multiple theoretical domains, across all capability-opportunity-motivation components. The discussion urges interventionists to consider the appropriateness of interventions in their development, feasibility/pilot, evaluation, and implementation stages.

**Registration:**

Prospero ID - CRD42019133735.

## Background

The World Health Organization describes hygiene practices as those “that help to maintain health and prevent the spread of diseases” [1]. These practices include behaviors to disinfect one’s body and surrounding environment [2]. Because bacteria that cause infection can live on one’s body and in the surrounding environment, preventing the spread of infectious diseases may require interventions that simultaneously encourage both personal- and environmental-disinfecting [3]. To improve public health many hand-hygiene interventions have been conducted in school-settings, wherein students may act as “agents of change” by carrying lessons about hygiene from school back to their home to influence family behavior [4–6]. The current systematic review was conducted to inform the development of future multifaceted interventions that aim to increase hand-hygiene and environmental-disinfecting behaviors in settings likely to include children.

Two recent systematic reviews closely informed the current review. The first is Willmott et al.’s 2016 review that included 18 school-based randomized controlled trials with hand-hygiene focused interventions [7]. The effectiveness of the interventions were assessed in terms of their ability to reduce negative health-related outcomes: absences and/or the spread of respiratory tract or gastrointestinal infections. The descriptions of the interventions suggest that most involved education/training (*N* = 15) and fewer involved infrastructural changes (*N* = 4). Only one study included measures of environmental-disinfecting (*N* = 1) and none included direct measures of hand-hygiene behavior (*N* = 0). Overall, they found equivocal evidence for the effectiveness of school-based interventions. However, as none of the studies directly measured hand-hygiene, it is uncertain whether they even influenced the process variable they were designed to most directly influence: hand-hygiene behavior. One of the effective interventions in this review took place in a childcare center, and this intervention simultaneously targeted hand-hygiene and environmental-disinfecting [8]. To this end, the current review aims to include studies that assess the effectiveness of interventions designed to improve hand-hygiene and/or environmental-disinfecting.

The second review that influenced the current review was conducted by Huis et al. in 2012 [9]. Huis et al.’s review included 41 intervention studies published between 2000 and 2009 to increase healthcare workers’ hand-hygiene compliance. In this review, the interventions were categorized according to the behavioral determinants that they were designed to influence [10, 11]. In so doing, this review brings together a wide range of interventions with a purposeful intervention terminology to guide future intervention development via the Behavior Change Wheel [12, 13]. The Behavior Change Wheel is a formal methodology that helps interventionists identify the most common reasons for sub-optimal behavior by providing a comprehensive list of empirically and theoretically informed reasons, e.g. lacking knowledge or resources to perform the desired behavior. The Behavior Change Wheel can be used as part of the first step in the Medical Research Council’s four-step Complex Intervention Development and Evaluation Framework. The steps include (1) Design, (2) Feasibility/piloting, (3) Evaluation and (4) Implementation [14]. This first step is important, because interventions designed to target uncommon reasons are unlikely to yield practically significant improvements.

Since Huis et al.’s review, the possible reasons for sub-optimal behavior have been more completely described in a taxonomy called the Theoretical Domain Framework (TDF) [15]. The TDF condenses 112 behavioral constructs into 14 domains that affect behavior: ‘Knowledge,’ ‘Behavioral Regulation,’ ‘Memory attention and decision processes,’ ‘Skills,’ ‘Goals,’ ‘Intentions,’ ‘Beliefs about consequences,’ ‘Beliefs about capabilities,’ ‘Optimism,’ Social/Professional role and identity,’ ‘Reinforcement,’ ‘Emotions,’ ‘Social influences,’ and ‘Environmental context and resources.’ These 14 domains are further condensed into the COM-B model’s three components, which exclusively and exhaustively explain why behaviors do or do not occur. The three COM-B components (and subcomponents) include Capability (physical/psychological), Opportunity (social/physical), and Motivation (reflective/automatic); the ‘B’ stands for Behavior. If even a single COM-B component is lacking, then a desired behavior is less likely to occur.

**Fig. 1:**
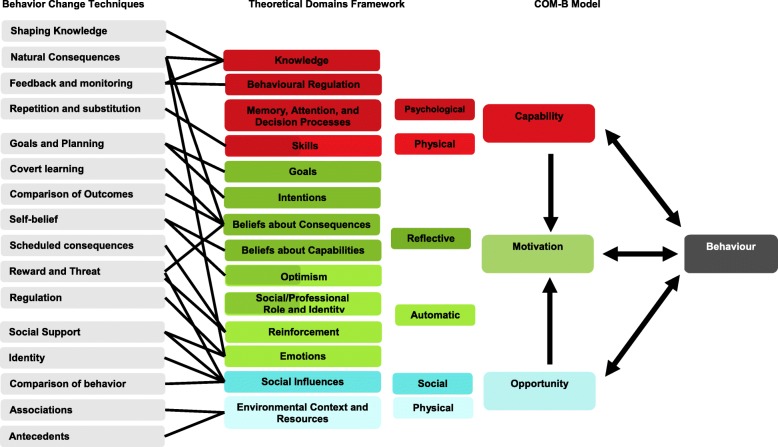
Links between the BCT clusters, TDF domains, and COM-B model

The TDF domains and COM-B model components are displayed in the second and third columns of Fig. 1. The links between them are indicated with shared colors, e.g. a dark red color is used to describe the link between the ‘Knowledge’ domain and the Capability-psychological component. After diagnosing the reasons for suboptimal behavior, the Behavior Change Wheel helps interventionists select the most appropriate intervention techniques. Ninety-three empirically and theoretically informed techniques are grouped into 16 clusters by the Behavior Change Techniques (BCTs) Taxonomy, version 1, e.g. *shaping knowledge, goals and planning, social support*, etc. [16]. In Fig. 1, the 16 BCT clusters are linked to their associated TDF domains by lines drawn across the first and second columns [17]. For example, the *shaping knowledge* technique is best suited to influence the ‘Knowledge’ domain.

Huis et al.’s 2012 review found that interventions targeting only one domain, e.g. only ‘Knowledge’ or only ‘Goals,’ were less effective than those that targeted multiple domains, e.g. ‘Knowledge’ and ‘Goals.’ Therefore, they suggest that future interventions should simultaneously target multiple domains, likely across the COM-B components, to increase optimal behavior. As the current review aims to influence the development of future multifaceted interventions, Huis et al.’s use of a purposeful intervention development terminology is desirable. Thus, the current review also categorizes interventions according to the techniques used and the domains/components targeted. In so doing, the current review will also guide future intervention development via the Behavior Change Wheel.

In summary, the current literature review was planned around two broad objectives. First, we aimed to learn what behavior change techniques had already been assessed to increase hand-hygiene and environment-disinfecting in settings likely to include children, e.g. schools, homes, etc. Second, where possible, we aimed to compare the effectiveness of these techniques and the domains/components they targeted. The discussion puts forth recommendations for the development of future multifaceted interventions.

## Methods

The current systematic review is reported in accordance with the Preferred Reporting Items for Systematic Review and Meta-Analysis (PRISMA) statement [18]. The review’s protocol was registered on 28th of May 2019 with PROSPERO: International Prospective Register of Systematic Review (Registration ID: CRD42019133735).

### Eligibility criteria

The eligibility criteria were determined using the PICO characteristics, i.e. characteristics describing the studies’ population, intervention(s), comparison(s) and outcome(s) [19]. The population characteristic was defined to include humans in settings likely to contain children less than 10-years-old and to exclude settings unlikely to contain children (e.g. manufacturing settings) and studies focused on non-human species. The intervention characteristic was defined to include studies that manipulated malleable factors likely to influence human behavior and to exclude comparisons of cleaning materials and non-malleable variables like gender. The comparison characteristic was defined to include any control or comparison condition, i.e. both randomized and pre-post observational trials. Finally, the outcome characteristic was defined to include hand-hygiene and environmental-disinfecting behavior measures. Environmental-disinfecting behavior was understood to entail the use of cleaning products to kill harmful germs that can cause illness.

### Information sources and search strategy

The search terms and selected databases were reviewed by the research team and library staff (Table 1). In addition to the search terms three inclusion criteria were applied. First, the articles had to be written in English, because no translation services were available to the research team. Second, the articles had to be published in peer-reviewed journals, to narrow the scope of the review to articles more likely to include relevant information. Third, the articles had to be published on or after January 2009. The final search was conducted on the 27th of April 2019 over EBSCOhost Medline and Web of Science Core Collection.

**Table 1:** PICO characteristics and search terms

PICO Characteristic	Search Terms
Population	(day-care OR “day care” OR childcare OR nursery OR school OR kindergarten OR student OR teacher OR child OR children OR parent OR community OR park OR playground OR home OR homes OR house OR household)
Intervention	behavio*
Comparison	(“randomized controlled trial” OR “randomised control trial” OR “randomized controlled trials” OR “randomised control trials” OR rct OR quasi-experimental OR observational OR “pre-test” OR pretest OR “post-test” OR posttest OR “crossover trial” OR “cross-over trial” OR intervention)
Outcome(s)	(wash OR washing OR hygiene OR clean* OR disinfect* OR sanitize OR sanitise OR soap)

### Study selection and data collection process

**Fig. 2:**
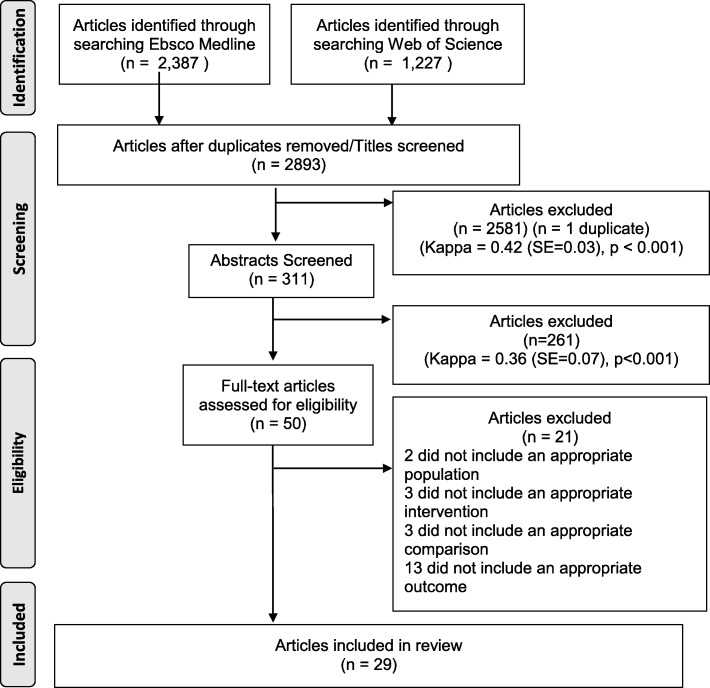
Prisma diagram describing how articles were located and screened

One researcher located the articles and then uploaded them to EndNote™ to combine, detect, and delete duplicate references. The remaining articles were uploaded to Rayyan QCRI [20]. Then, two researchers used Rayyan QCRI to independently screen titles and abstracts for inclusion. Full-text articles were collaboratively screened. The stages of the search and screening process are described in Fig. 2.

### Data extraction

Two reviewers extracted study data from the articles using data extraction questions first piloted on smaller samples of included studies. After the data extraction questions were finalized, each reviewer independently extracted data from approximately half of the included articles. The extracted data included study details, intervention descriptions, outcome descriptions, and findings. The interventions were defined according to the Behavioral Change Techniques Taxonomy, version 1 [16], and each technique’s cluster was linked to the theoretical domains and COM-B components, as described in Fig. 1 (also see Additional file 1 for the list of behavior change techniques and clusters); as discussed in the introduction, these links are informed by previous research [15–17]. The data extraction process was planned to permit a narrative summary of what types and numbers of behavioral domains and components were most likely to increase hand-hygiene and environmental-disinfecting.

### Overall quality assessment

One researcher reviewed articles to assess the studies’ overall quality using tools developed by the United States Department of Health and Human Services for controlled intervention and observational pre-post studies [21]. Each tool contains a checklist of items, e.g. asking about the sample-size and participant retention rates. To summarize the quality of the articles a five-star assessment was used. Four of the stars were assigned by taking the total number of positively indicated items divided by the total number of items: 1 star was given for positively indicating 25 to 49% of the items, 2 for 50 to 74%, 3 for 75 to 99%, and 4 stars for 100%. An additional star was given to those articles that use a randomized controlled trial methodology.

### Data synthesis

Narrative syntheses, with tallies, are used to summarize the findings. Tables are used to describe and aggregate summaries.

## Results

Of the 2893 titles assessed, 29 met the eligibility criteria (see Additional file 2). The reviewer agreements were moderate for screened titles (89.56%, Kappa = 0.42, *p* < 0.001) and abstracts (81.02%, Kappa = 0.36, *p* < 0.001). The studies took place mostly in Bangladesh (*N* = 6), and Kenya (*N* = 4). Fewer took place in India (*N* = 2), Peru (*N* = 2), South Africa (*N* = 2), the United States of America (*N* = 2), Zambia (*N* = 2), China (*N* = 1), Indonesia (*N* = 1), Iraq (*N* = 1), Laos (*N* = 1), Malawi (*N* = 1), Malaysia (*N* = 1), Nepal (*N* = 1), Tanzania (*N* = 1), and Zimbabwe (*N* = 1). Most of the studies were publically funded (*N* = 24).

### Study characteristics

Regarding the study designs, 7 were pre-post without randomization, 18 were pre-post with randomization, and the remaining 4 were randomized controlled trials with only post-intervention comparisons. Approximately one-third of the studies were pre-registered (*N* = 11). Nearly all of the studies indicated being granted approval by an ethics committee before commencing (*N* = 28). The remaining study did not indicate whether ethical approval was sought [22]. Prior to collecting data, a power analysis was conducted for most studies (*N* = 22), but this analysis was not always for an observable, behavioral measure, e.g. alternative primary outcomes included diarrhea episodes [23] and microbial counts [24]. Most of the interventions took place in schools (*N* = 12) or households with children (*N* = 13); fewer took place in pediatric settings (*N* = 2) or involved multiple locations, such as schools and other community centers or households (*N* = 2).

Regarding whose behavior was measured, 11 studies focused on the behavior of household members including children and adults, 16 focused on children/students, 1 looked at mother and child pairs [6], and 1 looked at pediatric healthcare workers [25]. Only 17 of the studies indicated the gender of their participants. In 23 studies some information about participants’ age was provided or could be inferred, e.g. from participants’ grade levels.

### Outcomes

All of the studies included a behavioral measure of hand-hygiene, but only 20 reported a significant increase in at least one measure of hand-hygiene, i.e. handwashing, handwashing with soap, or handwashing at key times (e.g. after defecation or before food preparation), compared to a control group or a pre-intervention measure. Nine interventions found no significant effect of the intervention condition on hand-hygiene. Only two studies included a measure of environmental-disinfecting, both were related to food preparation and both found significant increases. As so few articles were found for environmental-disinfecting, the remainder of the current results section focuses on hand-hygiene. In nearly half of the studies (*N* = 13), a health outcome measure was also recorded, such as absenteeism, diarrhea-symptoms, hospitalization episodes, and infection rates. The Additional file 3 provides details about the studies’ settings, participants, interventions, comparisons, outcome measurements, and results.

### Behavior change technique clusters and the COM-B model

The types and numbers of behavior change technique clusters (BCTs) employed are summarized in Table 2. Across the 29 studies the most commonly employed BCTs involved *shaping knowledge* (*N* = 22) and *antecedents* (*N* = 21). A moderate number of interventions involved *associations* (*N* = 14), *social support* (*N* = 12), *feedback and monitoring* (*N* = 10), *comparison of behaviors* (*N* = 8), and *goals and planning* (*N* = 7). Fewer interventions involved *repetition and substitution* (*N* = 5), *reward and threat* (*N* = 4), and *scheduled consequences* (*N* = 1) [26]. None of the interventions involved *comparison of outcomes*, *regulation*, *self-belief*, or *covert learning*. The interventions included as few as one BCT cluster [43–45] and as many as nine [34, 42]. Of the 29 included studies, 3 used a single BCT cluster, 15 included 2 to 4, and 11 included 5 or more. The mean number of BCT clusters per intervention that did not find a significant benefit for hand-hygiene was 3.00 (*SD* = 1.94, *Mdn* = 3). The mean number of interventions that did find a significant benefit was descriptively higher, i.e. 4.65 (*SD* = 2.30, *Mdn* = 4).

**Table 2:** The behavior change techniques reported alongside their associated theoretical domains, COM-B components, and whether significant benefits of the intervention were obtained

Behavior Change Technique^a^																	Total Number			Sig. Benefits	
Trials	1	2	3	4	5	6	7	8	9	10	11	12	13	14	15	16	Techniques	Domains	Components	HH^b^	ED^c^
1.Bieri et al. (2013) [26]	✔													✔			2	3	1	Yes	–
2.Biran et al. (2014) [27]	✔	✔	✔	✔								✔					5	7	3	Yes	–
3.Briceño et al. (2017) [23]		✔		✔	✔	✔						✔	✔				6	6	3	Yes	–
4.Bulled et al. (2017) [28]				✔	✔		✔					✔					4	4	3	Yes	–
5.Burns et al. (2018) [29]							✔			✔		✔					3	3	2	No	–
6.Caruso et al. (2014) [30]		✔		✔								✔					3	3	2	Yes	–
7.Chard et al. (2018) [31]			✔	✔	✔			✔				✔					5	6	3	No	–
8.Dreibelbis et al. (2016) [32]							✔					✔					2	1	1	Yes	–
9.Friedrich et al. (2018) [33]	✔	✔	✔	✔	✔		✔	✔				✔					8	9	3	Yes	–
10.Galliani et al. (2016) [22]			✔	✔													2	3	3	Yes	–
11.Gautam et al. (2017) [34]	✔	✔	✔	✔	✔	✔	✔			✔			✔				9	9	3	Yes	Yes
12.Geresomo et al. (2018) [35]			✔	✔	✔	✔						✔					5	4	3	Yes	Yes
13. Graves et al. (2012) [36]				✔			✔					✔					3	2	2	No	–
14.Greenland et al. (2016) [37]			✔	✔	✔	✔				✔							5	5	3	Yes	–
15.Grover et al. (2018) [38]				✔			✔					✔					3	2	2	Yes	–
16.Huda et al. (2012) [39]			✔	✔													2	3	3	No	–
17.Husain et al. (2018) [40]				✔	✔												2	3	3	No	–
18.Larson et al. (2018) [41]	✔	✔	✔	✔			✔					✔					6	7	3	Yes	–
19.Lewis et al. (2018) [6]	✔	✔		✔	✔		✔	✔				✔					7	8	3	No	–
20.Linam et al. (2011) [25]		✔	✔	✔								✔					4	5	3	Yes	–
21.Luby et al. (2018) [42]	✔	✔	✔	✔	✔	✔	✔			✔		✔					9	9	3	Yes	–
22.Naluonde et al. (2018) [43]							✔										1	1	1	No	–
23.Oswald et al. (2014) [44]												✔					1	1	1	No	–
24.Parvez et al. (2018) [45]												✔					1	1	1	Yes	–
25.Pickering et al. (2013) [46]				✔	✔	✔	✔	✔		✔		✔					7	7	3	Yes	–
26.Ram et al. (2017) [47]					✔		✔					✔					3	3	3	No	–
27.Saboori et al. (2013) [24]		✔		✔	✔							✔					4	5	3	Yes	–
28.Solehati et al. (2017) [48]			✔	✔		✔	✔										4	4	3	Yes	–
29.Watson et al. (2019) [49]				✔	✔	✔						✔					4	3	3	Yes	–

^a^ 1 = Goals and planning, 2 = Feedback and Monitoring, 3 = Social Support, 4 = Shaping knowledge, 5 = Natural consequences, 6 = Comparison of behaviors, 7 = Associations, 8 = Repetition and Substitution, 9 = Comparison of outcomes, 10 = Reward and threat, 11 = Regulation, 12 = Antecedents, 13 = Identity, 14 = Scheduled consequences, 15 = Self-belief, 16 = Covert learning

^b^ HH = Hand-Hygiene

^c^ ED = Environmental Disinfecting

Using the links provided in Fig. 1, the number of studies that targeted each TDF domain and COM-B component were tallied. The most frequently targeted domains were ‘Knowledge’ and ‘Environmental context and resources’ (both *N*’s = 22). Fewer studies targeted ‘Emotions’ (*N* = 20), ‘Beliefs in consequences’ (*N* = 15), ‘Social Influences’ (*N* = 14), ‘Behavioral Regulation’ (*N* = 10), ‘Goals’ (*N* = 7), ‘Intentions’ (*N* = 7), ‘Reinforcement’ (*N* = 5), ‘Skills’ (*N* = 4), and ‘Optimism’ (*N* = 1 [26];. No interventions targeted ‘Beliefs about capabilities.’ As a reminder no BCTs are linked to the ‘Memory attention and decision processes’ domain or ‘Social/Professional role and identity’ domain, and therefore it is not surprising that these domains were not targeted by any interventions. The studies targeted between 1 and 9 domains, with the average study targeting 4.38 domains (*SD* = 2.51, *Mdn* = 4). Of the 14 studies that targeted less than 4 domains, 7 (50%) found positive effects of the intervention. In contrast, of the 15 studies that targeted 4 or more domains, 13 (87%) found positive effects of the intervention.

Regarding the COM-B model, almost all the studies targeted Capability (*N* = 28), and many targeted Opportunity (*N* = 24) and Motivation (*N* = 21). Five of the studies only targeted one component, of which four targeted Opportunity and one targeted Motivation; only three of these five studies (60%) found a significant benefit. Four of the studies only targeted two components, of which three targeted Capability and Opportunity and one targeted Motivation and Opportunity; only two of these studies (50%) found a significant benefit. The remaining 20 studies targeted all three COM-B components, and 15 of these studies (75%) found a significant benefit.

### Methodological quality

The quality assessment for each study is provided in Additional file 4. As a reminder the studies were assessed with five stars, where four stars were allocated based on the percentage of assessment criteria met, and one star was added to studies that used a randomized controlled trial methodology. Of the 29 studies included, 2 studies received one star [36, 44], 21 received three stars, 2 received four stars [6, 43], and 4 received two stars [25, 27, 32, 33].

### Synthesis of results

The co-authors agreed that a pooled estimate of the effects would be misleading, due to the heterogeneity of the populations examined, research methods employed, and outcomes measured.

## Discussion

The current systematic review located 29 studies with interventions designed to increase hand-hygiene in settings likely to include children. Of the 29 studies, only 2 were also designed to increase environmental-disinfecting behavior. Individual study results suggest that interventions may increase hand-hygiene and environmental-disinfecting, but the behavior change techniques they employed and domains/components they targeted varied. The most targeted domains were ‘Knowledge’ and ‘Environmental context and resources.’ Descriptively, interventions targeting four or more theoretical domains and those targeting all the COM-B components were more likely to succeed.

The findings of this literature review align with other reviews emphasizing the value of multifaceted interventions. As stated in the introduction, the COM-B model proposes that people need sufficient Capability, Opportunity, and Motivation to perform a desired behavior. If even a single component is lacking, then people will be less likely to perform the desired behavior [12]. Agreeing with the COM-B framework, Harvey and Kitson argue that interventions meant to influence a greater range of people with more complex problems often require multifaceted approaches [50]. As hand-hygiene is likely a complex behavioral problem, interventions designed to affect a single component may prove inadequate to produce either population-level benefits (as individuals experience different barriers) or individual-level benefits (as each individual experiences multiple barriers that need to be simultaneously overcome).

Comparing interventions designed to affect each TDF domain or COM-B component, in isolation and combination, would help interventionists better understand how these domains/components influence each other. However, such factorial experimental designs will prove difficult to conduct given real-world constraints. Further the scientific exactness of factorial designs are likely outside the scope of many studies with more practical aims. In many studies, hand-hygiene is operationalized as a process variable (that may or may not be measured) meant to impact a health outcome (that is measured), and previous systematic reviews have largely focused on practical health outcomes. For example, Willmott et al.’s (2016) review located 18 randomized controlled trials that investigated the effectiveness of hand-hygiene interventions on children’s absences and infections [7]; Meadows et al.’s (2004) review located 6 studies evaluating the effectiveness of antimicrobial rinse-free hand sanitizer interventions on elementary school children’s absenteeism due to communicable illness [51]; and Wilson et al.’s (2006) located 12 studies that investigated the effectiveness of hand-hygiene interventions to decrease infections and absenteeism [52].

Studies focusing on hand-hygiene behavior itself are likely more common in health care settings [53–55], where hand-hygiene compliance audits are already common. In contrast, in school-settings hand-hygiene compliance audits may prove difficult to fund, develop, and faithfully implement. As a result of these difficulties, interventions in school settings are often evaluated using the data that schools already regularly collect, e.g. absences, or that parents/students can self-report with reasonable face-validity, e.g. diarrhea episodes. While outcomes like absences and diarrhea episodes are certainly important, the present research team argues that there is already sufficient evidence that hand-hygiene impacts these health outcomes [56, 57]. Therefore, more studies and reviews looking at the effectiveness of hand-hygiene interventions should prioritize observable hand-hygiene behavior measures when assessing their interventions’ effects.

### Limitations

Several limitations of the current review will now be acknowledged. First, the search only included two data-bases, articles published in the English language, and did not extend to the grey literature. Given the current research team’s time and resource constraints, these restrictions were necessary. A future review aiming to understand what techniques have been attempted (with or without being assessed) may find it useful to include the grey literature. Another limitation of the review is its rigid focus on observable behaviors. Indeed, most studies discarded from the review during the full-text screening were lost because they did not include measures of observable behavior, but rather only included self-reported measures.

### Recommendations for future intervention studies

The current review recommends that future interventions designed to increase hand-hygiene or environmental-disinfecting in settings likely to include children target multiple theoretical domains and all COM-B components. Which domains are targeted will depend on the particular setting and population. For example, if the particular setting already includes sufficient infrastructure for children to carry out hand-hygiene, e.g. soap and a water basin, then providing more soap or installing new water basins is unlikely to produce a beneficial effect; though, making children aware of such materials might. The only way to be more certain about what barriers a particular population experiences is to conduct formative research in the selected setting with the selected population, e.g. structured observations, focus groups, interviews, surveys, etc. Such formative research should aim to comprehensively examine all the possible barriers that could influence hand-hygiene, because if even a single component is lacking, then beneficial effects of the intervention are less likely to be realized. The Behavior Change Wheel can be used to guide the development of multifaceted interventions, and the selection of the most appropriate intervention functions (e.g. education or persuasion) and policy categories (e.g. guidelines or legislation) through which those interventions can be delivered [58].

Of course, selecting behavior change techniques is only part of the intervention development process. Beyond targeting the right barriers, the intervention must be implemented through an appropriate mode. To bolster the appropriateness of the ultimate intervention, interventionists can use the APEASE criteria [59]. APEASE is an acronym in which each letter stands for a different appropriateness-criterion: Affordability, Practicality, Effectiveness, Acceptability, Side-effects, and Equity. A sample of questions researchers might ask themselves about each criterion are provided in Table 3. The APEASE criteria should be consulted iteratively during an intervention’s development, feasibility/pilot testing, evaluation, and implementation [58]. Considering the APEASE criteria during the development phase is important; if stakeholders do not believe the intervention is appropriate, then the intervention will prove difficult to scale and spread even if the intervention’s effects are found to be beneficial.

**Table 3:** The APEASE criteria and example questions

Criteria	Example Questions
Affordable	Would others be able and willing to pay to implement the intervention?
Practicability	Would others have sufficient physical resources or sufficiently trained staff to implement the intervention?
Effectiveness	Would others believe the likely effect-size of the intervention was sufficient to justify the time and resources necessary to implement it?
Acceptability	Would relevant stakeholders (public, professional, and political) deem the intervention socially appropriate?
Side-effects	What side-effects (positive or negative) are likely to emerge and how could they be monitored? How can potential negative side-effects be mitigated?
Equity	Will the intervention increase unwanted disparities in different people’s standard of living, psychological wellbeing, or physical wellbeing?

The present review focused on the behavior change techniques, theoretical domains, and COM-B components interventionists should consider when developing a multifaceted intervention. After developing a multifaceted intervention, the Behavior Change Wheel and the Medical Research Council’s Complex Intervention Development and Evaluation Framework recommend feasibility/pilot testing [12, 14]. Specific information regarding how to feasibility/pilot test an intervention study is outside the scope of the present review. Briefly here, note that while one may be uncertain about the benefits of a intervention before full-scale testing, feasibility/pilot tests help one to become more certain about the parameters needed for a fair full-scale test of that intervention’s effectiveness. Many, often costly, trials that do not first feasibility/pilot test their interventions ultimately fail to find significant effects due to factors that better planning may have mitigated, e.g. the sample-size was too low, people found the intervention unacceptable, or intervention implementation was inadequate [60–62].

## Conclusions

The current literature review identified 29 studies with interventions that aimed to increase hand-hygiene, 2 of which also aimed to increase environmental-disinfecting. In alignment with previous findings, this review finds that interventions that simultaneously target more theoretical domains and all COM-B components are descriptively more likely to succeed. The review also notes that very few trials examine hand-hygiene and environmental-disinfecting simultaneously and encourages more studies to do so, as this may be the most cost-effective way to halt reinfection cycles. In the discussion, interventionists were urged to consider the appropriateness of their interventions in the development, feasibility/pilot, evaluation, and implementation stages. This iterative and methodical process can encourage better scale and spread of effective interventions that increase hand-hygiene and environmental-disinfecting behaviors in settings likely to include children.

## Supplementary information

**Additional file 1.** Links between the Theoretical Domains and Behavior Change Technique (version 1) used in the current research project.

**Additional file 2.** References for articles included in the review.

**Additional file 3.** Characteristics of included studies in the systematic review.

**Additional file 4.** Quality assessments conducted using the United States Department of Health and Human Services tool for pre-post studies.

## Data Availability

The reviews protocol is available on PROSPERO. The datasets used and/or analysed during the current study are available from the corresponding author on reasonable request.

## Abbreviations

CG: Control Group
ED: Environmental Disinfecting
F: Female
HH: Hand
HW: Handwashing
HWWS: Handwashing with soap
IG: Intervention Group
M: Male
TDF: Theoretical Domains Framework
