# Supplementary material for: A systematic review of hand-hygiene and environmental-disinfection interventions in settings with children
Source: BMC Public Health. 2020 Feb 6;20:195. doi: 10.1186/s12889-020-8301-0 (PMC7006391; doi:10.1186/s12889-020-8301-0)
Supplement: Supplementary file 1 — Additional file 1. Links between the Theoretical Domains and Behavior Change Technique (version 1) used in the current research project. [file 12889_2020_8301_MOESM1_ESM.docx]

Additional file 1 Links between the Theoretical Domains and Behavior Change Technique (version 1) used in the current research project.

| TDF Domain^a^ | Behavior Change Technique^b^ | |  |
| --- | --- | --- | --- |
| Intentions/ Goals | | **1. Goals and Planning** | |
|  | | 1.1. Goal setting (behavior) | |
|  | | 1.2. Problem solving | |
|  | | 1.3. Goal setting (outcome) | |
|  | | 1.4. Action planning | |
|  | | 1.5 Review behavior goal(s) | |
|  | | 1.6. Discrepancy between current behavior and goal(s) | |
|  | | 1.7. Review outcome goal(s) | |
|  | | 1.8. Behavioral contract | |
|  | | 1.9. Commitment | |
| Knowledge/ Behavioral regulation | | **2. Feedback and monitoring** | |
|  | | 2.1 Monitoring of behavior by others without feedback | |
|  | | 2.2. Feedback on behavior | |
|  | | 2.3. Self-monitoring of behavior | |
|  | | 2.4. Self-monitoring of outcome(s) of behavior | |
|  | | 2.5. Monitoring of outcome(s) of behavior without feedback | |
|  | | 2.6. Biofeedback | |
|  | | 2.7. Feedback on outcome(s) of behavior | |
| Social Influence / Emotions | | **3. Social Support** | |
|  | | 3.1. Social support (unspecified) | |
|  | | 3.2. Social support (practical) | |
|  | | 3.3. Social support (emotional) | |
| Knowledge | | **4. Shaping knowledge** | |
|  | | 4.1. Instructions on how to perform the behavior | |
|  | | 4.2. Information about antecedents | |
|  | | 4.3. Re-attribution | |
|  | | 4.4. Behavioral experiments | |
| Knowledge /Emotions/ Beliefs about consequences | | **5. Natural consequences** | |
|  | | 5.1. Information about health consequences | |
|  | | 5.2. Salience of consequences | |
|  | | 5.3. Information about social and environmental consequences | |
|  | | 5.4. Monitoring of emotional consequences | |
|  | | 5.5. Anticipated regret | |
|  | | 5.6. Information about emotional consequences | |
| Social Influences | | **6. Comparison of behavior** | |
|  | | 6.1. Demonstration of the behavior | |
|  | | 6.2. Social comparison | |
|  | | 6.3. Information about others' approval | |
| Environmental context and resources | | **7. Associations** | |
|  | | 7.1. Prompts/cues | |
|  | | 7.2. Cue signalling reward | |
|  | | 7.3. Reduce prompts/cues | |
|  | | 7.4. Remove access to the reward | |
|  | | 7.5. Remove aversive stimulus | |
|  | | 7.6. Satiation | |
|  | | 7.7. Exposure | |
|  | | 7.8. Associative learning | |
| Skills | | **8. Repetition and substitution** | |
|  | | 8.1. Behavioral practice/ rehearsal | |
|  | | 8.2. Behavioral substitution | |
|  | | 8.3. Habit formation | |
|  | | 8.4. Habit reversal | |
|  | | 8.5. Overcorrection | |
|  | | 8.6. Generalization of target behavior | |
|  | | 8.7. Graded tasks | |
| Beliefs about consequences | | **9. Comparison of outcomes** | |
|  | | 9.1. Credible source | |
|  | | 9.2. Pros and cons | |
|  | | 9.3. Comparative imagining of future outcomes | |
| Reinforcement / Beliefs about Consequences/ Social Influences | | **10. Reward and threat** | |
|  | | 10.1. Material incentive (behavior) | |
|  | | 10.2. Material reward (behavior) | |
|  | | 10.3. Non-specific reward | |
|  | | 10.4. Social reward | |
|  | | 10.5. Social incentive | |
|  | | 10.6. Non-specific incentive | |
|  | | 10.7. Self-incentive | |
|  | | 10.8. Incentive (outcome) | |
|  | | 10.9. Self-reward | |
|  | | 10.10. Reward (outcome) | |
|  | | 10.11. Future punishment | |
| Emotions | | 1. **Regulation** | |
|  | | 11.1. Pharmacological support | |
|  | | 11.2. Reduce negative emotions | |
|  | | 11.3. Conserving mental resources | |
|  | | 11.4. Paradoxical instructions | |
| Environmental context and resources | | **12. Antecedents** | |
|  | | 12.1 Restructuring the physical environment | |
|  | | 12.2. Restructuring the social environment | |
|  | | 12.3. Avoidance/ reducing exposure to cues for the behavior | |
|  | | 12.4. Distraction | |
|  | | 12.5. Adding objects to the environment | |
|  | | 12.6. Body changes | |
| Social Influences | | **13. Identity** | |
|  | | 13.1. Identification of self as a role model | |
|  | | 13.2. Framing/reframing | |
|  | | 13.3. Incompatible beliefs | |
|  | | 13.4. Valued self-identity | |
|  | | 13.5. Identity associated with changed behavior | |
| Reinforcement | | **14. Scheduled consequences** | |
|  | | 14.1. Behavior cost | |
|  | | 14.2. Punishment | |
|  | | 14.3. Removed reward | |
|  | | 14.4. Reward approximation | |
|  | | 14.5. Rewarding completion | |
|  | | 14.6. Situation-specific reward | |
|  | | 14.7. Reward incompatible behavior | |
|  | | 14.8. Reward alternative behavior | |
|  | | 14.9. Reduce reward frequency | |
| Beliefs about capabilities/ Optimism | | **15. Self-belief** | |
|  | | 15.1. Verbal persuasion about capability | |
|  | | 15.2. Mental rehearsal of successful performance | |
|  | | 15.3. Focus on past success | |
|  | | 15.4. Self-talk | |
| Beliefs about consequences | | **16. Covert learning** | |
|  | | 16.1. Imaginary punishment | |
|  | | 16.2. Imaginary reward | |
|  | | 16.3. Vicarious consequences | |

^a^Links between the Domains and Techniques were taken from Table 5 in Cane et al. (2015) [17]

^b^The hierarchy of Behavior Change Techniques (version 1) and were taken from Michie et al.’s (2013, pp. 11-30) Supplementary Materials, where the definition of each technique is located. [16]
