# Supplementary material for: A systematic review of hand-hygiene and environmental-disinfection interventions in settings with children
Source: BMC Public Health. 2020 Feb 6;20:195. doi: 10.1186/s12889-020-8301-0 (PMC7006391; doi:10.1186/s12889-020-8301-0)
Supplement: Supplementary file 2 — Additional file 2. References for articles included in the review. [file 12889_2020_8301_MOESM2_ESM.docx]

Additional file 2 References for articles included in the review.

1. Bieri FA, Gray DJ, Williams GM, Raso G, Li Y-S, Yuan L, et al. Health-education package to prevent worm infections in Chinese school children. *N Engl J Med.* 2013;368(17):1603-1612. doi:10.1056/NEJMoa1204885

2. Biran A, Schmidt W-P, Varadharajan KJ, Rajaraman D, Kumar R, Greenland K, et al. Effect of behavior-change intervention on handwashing with soap in India (SuperAmma): a cluster-randomised trial. Lancet Glob Health. 2014;2(3):e145-154.

3. Briceño B, Coville A, Gertler P, Martinez S. Are there synergies from combining hygiene and sanitation promotion campaigns: evidence from a large-scale cluster-randomized trial in rural Tanzania. PloS. 2017;12(11):e0186228.

4. Bulled N, Poppe K, Ramatsisti K, Sitsula L, Winegar G, Gumbo J, et al. Assessing the environmental context of hand washing among school children in Limpopo, South Africa. Water International. 2017;42(5):568-584. doi:10.1080/02508060.2017.1335140

5. Burns J, Maughan-Brown B, Mouzinho A. Washing with hope: evidence of improved handwashing among children in South Africa from a pilot study of a novel soap technology. BMC Public Health. 2018;18(1):709. doi:10.1186/s12889-018-5573-8

6. Caruso BA, Freeman MC, Garn JV, Dreibelbis R, Saboori S, Muga R, Rheingans R. Assessing the impact of a school-based latrine cleaning and handwashing program on pupil absence in Nyanza Province, Kenya: a cluster-randomized trial. Trop Med Int Health. 2014;19(10):1185-1197. doi:10.1111/tmi.12360

7. Chard AN, Freeman MC. Design, intervention fidelity, and behavioral outcomes of a school-based water, sanitation, and hygiene cluster-randomized trial in Laos. Int J Environ Res Public Health. 2018;15(4):570. doi:10.3390/ijerph15040570

8. Dreibelbis R, Kroeger A, Hossain K, Venkatesh M, Ram PK. Behavior Change without Behavior Change Communication: nudging Handwashing among Primary School Students in Bangladesh. Int J Environ Res Public Health. 2016;13(1):129. doi:10.3390/ijerph13010129

9. Friedrich MND, Kappler A, Mosler H. Enhancing handwashing frequency and technique of primary caregivers in Harare, Zimbabwe: a cluster-randomized controlled trial using behavioral and microbial outcomes. Soc Sci Med. 2018;196:66-76. doi:10.1016/j.socscimed.2017.10.025

10. Galliani S, Gertler P, Ajzenman N, Orsola-Vidal A. Promoting handwashing behavior: The Effects of large-scale community and school level interventions*.* Health Econ. 2016:25(12):1545-1559. doi:10.1002/hec.3273

11. Gautam OP, Schmidt WP, Cairncross S, Cavill S, Curtis V. Trial of a novel intervention to improve multiple food hygiene behaviors in Nepal. Am J Trop Med Hyg. 2017;96(6):1415-1426. doi:10.4269/ajtmh.16-0526

12. Geresomo NC, Mbuthia EK, Matofari JW, Mwangwela AM. Targeting caregivers with context specific behavior change training increased uptake of recommended hygiene practices during food preparation and complementary feeding in Dedza district of Central Malawi. Ecology of Food and Nutrition. 2018;57(4):301-313. doi:10.1080/03670244.2018.1492379

13. Graves JM, Daniell WE, Harris JR, Obure AFXO, Quick R. Enhancing a safe water intervention with student-created visual aids to promote handwashing behavior in Kenyan primary schools. Int Q Community Health Educ. 2012;32(4):307-323. doi:10.2190/IQ.32.4.d

14. Greenland K, Chipungu J, Curtis V, Schmidt WP, Siwale Z, Mudenda M, et al. Multiple behavior change intervention for diarrhoea control in Lusaka, Zambia: a cluster randomised trial. Lancet Glob Health, 2016;4(12):e966-e977. doi:10.1016/S2214-109X(16)30262-5

15. Grover E, Hossain MK, Uddin S, Venkatesh M, Ram PK, Dreibelbis R. Comparing the behavioral impact of a nudge based handwashing intervention to high intensity hygiene education: a cluster-randomised trial in rural Bangladesh. Trop Med Int Health. 2018;23(1):10-25. doi:10.1111/tmi.12999

16. Huda TN, Unicomb L, Johnston RB, Halder AK, Sharker AY, Luby SP. Interim evaluation of a large scale sanitation, hygiene and water improvement programme on childhood diarrhea and respiratory disease in rural Bangladesh. Soc Sci Med. 2012;75:604-611. doi:10.1016/j.socscimed.2011.10.042

17. Husain N, Mankan W, Izani N, Nurain N, Razlina AR. The effect of food safety education on handwashing practices in school canteens food handlers. Sains Malaysiana. 2018;47(9):2119-2128.

18. Larson EL, Murray MT, Cohen B, Simpser E, Pavia M, Jackson O, et al. Behavioral interventions to reduce infections in pediatric long-term care facilities: the Keep it Clean for Kids trial. Behav Med. 2018;44(2):141-150. doi:10.1080/08964289.2017.1288607

19. Lewis HE, Greenland K, Curtis V, Schmidt WP. Effect of a school-based hygiene behavior change campaign on handwashing with soap in Bihar, India: cluster-randomized trial. Am J Trop Med Hyg. 2018:99(4):924-933. doi:10.4269/ajtmh.18-0187

20. Linam MW, Margolis PA, Atherton H, Connelly BL. Quality-improvement initiative sustains improvement in pediatric health care worker hand-hygiene. Pediatrics. 2011:128:e689-698, doi:10.1542/peds.2010-3587

21. Luby SP, Kadir M, Sharker MAY, Yeasmin F, Unicomb L, Islam MS. A community-randomised controlled trial promoting waterless hand sanitizer and handwashing with soap, Dhaka, Bangladesh. Trop Med Int Health. 2010:15(12):1508-1516. doi:10.1111/j.1365-3156.2010.02648.x

22. Naluonde T, Wakefield C, Markle L, Martin A, Tresphor C, Abdullah R, Larsen DA. A disruptive cue improves handwashing in school children in Zambia. Health Promot Int. 2018:1-10.

23. Oswald WE, Hunter GC, Kramer MR, Leontsini E, Cabrera L, Lescano AG, Gilman RH. Provision of private, piped water and sewerage connections and directly observed handwashing of mothers in a peri-urban community of Lima, Peru. Trop Med Int Health, 2014;19(4):388-397. doi:10.1111/tmi.12262

24. Parvez SM, Azad R, Rahman M, Unicomb L, Ram PK, Naser AM, et al. Achieving optimal technology and behavioral uptake of single and combined interventions of water, sanitation hygiene and nutrition, in an efficacy trial (WASH benefits) in rural Bangladesh. BMC Trials. 2018;19:358. doi:10.1186/s13063-018-2710-8

25. Pickering AJ, Davis J, Blum AG, Scalmanini J, Oyier B, Okoth G, et al. Access to waterless hand sanitizer improves student hand-hygiene behavior in primary schools in Nairobi, Kenya. Am J Trop Med Hyg. 2013;89(3):411-418. doi:10.4269/ajtmh.13-0008

26. Ram KP, Nasreen S, Kamm K, Allen J, Kumar S, Rahman MA, et al. Impact of an intensive perinatal handwashing behavior in neonatal period: findings from a randomized controlled trial in rural Bangladesh. Hindawi Biomedical Research International, 2017;6081470. doi:10.1155/2017/6081470

27. Saboori S, Greene LE, Moe CL, Freeman MC, Caruso BA, Akoko D, Rheingans RD. Impact of regular soap provision to primary schools on hand washing and E. coli hand contamination among pupils in Nyanza Province, Kenya: a cluster-randomized trial. Am J Trop Med Hyg. 2013:89(4):698-708. doi:10.4269/ajtmh.12-0387

28. Solehati T, Kosasih CE, Susilawati S, Lukman M, Paryati, SPY. Effect of school community empowerment model towards handwashing implementation among Elementary School Students in Dayeuhkolot Subdistrict, Kesmas. National Public Health Journal. 2017;11(3):111-116. doi:10.21109/kesmas.v11i3.1171

29. Watson J, Dreibelbis R, Aunger R, Deola C, King K, Long Sl, et al. Child's play: harnessing play and curiosity motives to improve child handwashing in a humanitarian setting. Int J Hyg Environ Health. 2019;*222*(2):177-182. doi:10.1016/j.ijheh.2018.09.002
