# Supplementary material for: A systematic review of hand-hygiene and environmental-disinfection interventions in settings with children
Source: BMC Public Health. 2020 Feb 6;20:195. doi: 10.1186/s12889-020-8301-0 (PMC7006391; doi:10.1186/s12889-020-8301-0)
Supplement: Supplementary file 3 — Additional file 3. Characteristics of included studies in the systematic review. [file 12889_2020_8301_MOESM3_ESM.docx]

Additional file 3 Characteristics of included studies in the systematic review

| Author, trial design | Setting | Participants, Gender, Age | Intervention description^a^ | Comparator condition | Description of HW* and /or ED* measure and frequency of measure | Intervention effect | Quality rating |
| --- | --- | --- | --- | --- | --- | --- | --- |
| 1.Bieri et al. (2013), RCT [26] | 38  schools  China | 1934 students,  M*=976, F*=739,  Age=10-13 | One IG* experienced health education focused posters (4.0) displayed in schools alongside educational material on the transmission and prevention of soil transmitted helminths – Reinforced via built in competitions (14.0). | Control schools received health education poster only | Trained researchers completed structured observations covertly in morning and lunch breaks - focused on students HW after toilet use measured at baseline and post intervention. | Significant difference in HW after toilet use in intervention vs control schools. | ★★★ |
| 2.Biran et al. (2014),  RCT [27] | 14  villages  India | 2183 villagers,  Gender=NA,  Age=NA | One IG experienced a goals and planning intervention (1.0) along with provision of feedback and monitoring of HW behaviors (2.0), a social support network promoting HW (3.0), education around HH* (4.0) and provisions of HW resources to allow participants to carry out HW (12.1). | No treatment CG* | Female observers in households from 05.30-08.30am - observed household members HWWS* after key events (after defecation, after cleaning a child’s bottom, before food preparation and before eating).  Measured at baseline, 6 weeks, 6 months and 12 months (4 time points). | Significant difference in HWWS at key time points. | ★★★ |
| 3.Briceño et al. (2017),  RCT [23] | 181  rural wards  Tanzania | 724 primary caregivers with 1 child <5, 1 school aged child  F=724  Age=NA | Three IGs called the HWWS intervention, the Sanitation Intervention, and HWWS + Sanitation Interventions Combined.  HWWS Intervention: Over about 1 year, a social marketing intervention was used to influence mothers with children <5 years old, by tapping into their social identity as hard working mothers. It focused on raising awareness of the importance of HWWS (5.1) and providing technical assistance to build HW stations with local materials (4.1). Local campaign workers were trained for 3 days (4.1) to conduct household visits and spread the campaign messages and distributed promotional materials at market days, pre-natal clinics and village meetings (12.5). The campaign message was also spread in traveling roadshows and on radio shows.  Sanitation Intervention: Over about 1 year, a social marketing intervention was used to influence the heads of households to promote hygienic latrines. Local campaign workers were trained in each ward to set up a latrine register to monitor their community’s progress (2.1, 2.5) and to continue to motivate households to build clean latrines (3.1, 6.2, 6.3). In addition, masons were taught to construct cement sanitation platforms (4.1) that they could sell at a reasonable price (12.5).  HWWS +Sanitation Interventions Combined: See previous information. | No treatment CG | 3-hour structured observations post intervention (pre survey to avoid Hawthorne effect). Measured whether the caregiver used water and soap to wash their own or their child's hands at key exposure events: 1) After fecal contact: (i) after defecating; (ii) after toileting; (iii) after cleaning child post toileting 2) Before handling food: (i) before cutting or preparing food; (ii) before eating; (iii) before serving food; (iv) before breastfeeding. Observers also scored hand cleanliness. | Significant effect on HH in HH only intervention, HH + sanitation intervention and no significant effect in sanitation only condition. | ★★★ |
| 4.Bulled et al. (2017),  Observational pre-post measurements  [28] | 2 schools  South Africa | 600 students,  Gender=NA,  Age=6-13 | Two IGs called the Infrastructure/HWWS intervention and the HWWS intervention. Infrastructure/HWWS intervention: School A’s environment was restructured to position 'taps' closer to the toileting facilities and to increase the number of stalls (12.1). Bars of soap were also made available (12.5). In addition to the structural improvements, posters were displayed in the toileting facilities and classrooms about the three HW steps: rise, lather, rinse, and the lyrics to a HW song (4.1) were taught and posted to remind students to wash properly (7.1). Also a multi-dimensional education program was taught to students on HW (4.1) that told them why they should (5.1). 30-minute school assemblies emphasized the communal benefits to good HH (5.3) and students performed plays to demonstrate how illness spreads (5.1).  HWWS Intervention: School B had HW posters displayed in the classroom plus lyrics to a HW song (4.1) to remind students to wash properly (7.1). In addition a multi-dimensional education program that taught students to wash their hands (4.1), told them why they should (5.1), 30-minute school assemblies emphasized the communal benefits to good HH (5.3) and students performed plays to demonstrate how illness spreads (5.1). | No CG | Structured observations by observers of HW after toilet use and HW not linked to toilet use. Recorded student counts i.e. how many students washed their hands after toileting. Observations over 2 consecutive days, during 2 break periods when most students utilized the sanitation facilities (9:30am – 10:00am and 12:00pm – 12:10pm).  At School A, all measurements were taken pre, post-environmental intervention, and post-social intervention. At School B, all measurements were taken pre, and post-social intervention. | Significant effect on HW after toilet use and independent of toilet use after social interventions in school A and B.  HW independent of toilet use after environmental intervention school A. | ★★ |
| 5.Burns et al. (2018),  RCT [29] | 203 households  South Africa | 229 Caregivers and  287 children,  Gender=NA  Children’s Age=3-9 | One IG received soap with toy inside to encourage goal oriented HW in the children through receipt of material reward for the performance of the behavior (10.2). The toy soaps were delivered fortnightly by an independent team over a 6 week period. | Soap only + toy separate CG | Snack test = community worker observed whether child washed hands unprompted after they gave them a snack in 4 scheduled home visits. | No significant difference in unprompted HW after snack in IG vs CG (6% percentage point greater likelihood of HW reported in IG vs CG at post intervention= non-significant). | ★★★ |
| 6.Caruso et al. (2014),  RCT [30] | 60  schools  Kenya | 17,564 students  CG Grades1-8= 5302 (2615 girls)  HW arm: Grades 1-8= 5490 (2674= girls)  HW + latrine cleaning arm: Grades 1-8=6772 (3319 girls) | Two IGs called the HW Intervention and the HW + Latrine cleaning intervention.  HW Intervention: Teachers were trained on the correct HW procedures (4.1) supported by restructuring of physical environment and the provision of soap and HW material (12.1).  HW + Latrine cleaning: Teachers were trained on correct HW procedures (4.1) supported by restructuring of the physical intervention environment and provision of soap and HW materials (12.1). In addition, teachers were trained for correct latrine cleaning procedures (4.1), latrine conditions monitored and feedback provided (2.0). | No treatment CG | Structured observations by enumerators over 30 minute period in morning recess. Recorded children HW and HWWS at baseline and post intervention (reported as an average over the follow-up rounds). | Significant differences between IG and CG for HWWS and any type of HW behavior. | ★★★ |
| 7.Chard et al. (2018),  RCT [31] | 100  schools  Laos | Total number of students NA,  Gender=NA,  Age= Primary school | Two IGs called the Initial Intervention and Enhanced Intervention.  Initial Intervention (group 1 intervention schools): Schools were provided with a water supply connected to 3 toilet compartments and 2 sinks, classrooms received a ceramic water filter maintained and filled with water by teachers (12.1, 12.4). Teachers lead group HH practice 2 times each day (4.1, 5.1, 8.1). Student teams performed light routine cleaning and maintenance plus school compound maintenance (3.2, 12.2).  Enhanced Intervention (group 2 intervention schools): Adjustments to the initial intervention were made to ensure that more schools would have an appropriate water supply for HW (12.1, 12.5). | No treatment CG | Enumerators completed structured observations of:  1) HH Percent – recorded who uses toilet and whether they use soap and water after exiting.  2) Daily Group HW: record whether schools conduct group HW activities. On average, data were collected (over 2 years) following hardware completion in Group 1 schools, and 5 visits (over 1.25 years) following hardware completion in Group 2 schools. | No significant effect reported for initial intervention on HH percentage yet significant effect for the enhanced intervention. Significant effect reported on both initial and enhanced intervention for daily group HW. | ★★★ |
| 8.Dreibelbis et al. (2016),  Observational, pre-post measurements [32] | 2  schools  Bangladesh | 734 students,  Gender=NA,  Age= Primary school aged | The intervention was rolled out over three phases, called Infrastructure Intervention, Footpath Only Intervention, and Footpath + Footprint Paintings Intervention.  Infrastructure Intervention: In 1 day, soap was provided to the schools and a dedicated location for HWWS was constructed (12.5).  Footpath Only Intervention: In the same schools, in one day, a footpath was constructed connecting the latrines to the HW facilities (12.5).  Footpaths and Footprint Paintings Intervention: In the same schools, in 1 day, footprints were painted on the footpaths prompting students to walk to the HW facilities (7.1). | No CG | Staff completed structured observations (over 1 school day 8-3pm) recording HH percent noting children HW and HWWS  6 observations were completed at baseline (prior to construction), 1 day after each infrastructure and nudge installation (HW infrastructure, brick paths and painting) and 2 and 6 weeks after the intervention was completed. | Significant effect on HH percentage. | ★★ |
| 9.Friedrich et al. (2018),  RCT [33] | 20  high density community areas  Zimbabwe | 600 primary caregivers of a child attending school,  F=582, M=18,  Age=NA | Three IGs called the Direct HWWS Intervention in Communities, the Indirect HWWS Intervention in Schools, and the Combined Intervention. Direct HWWS Intervention in Communities: Over 1 week, local health center staff talked with community members about health and emotional consequences of HWWS (3.2, 4.2, 5.1, 5.6), practiced the HW steps (8.1), helped community members develop an action plan to wash their hands (1.4), provided prompts /like posters - to help people remember to wash hands (7.1), asked people to self-monitor their HW on a given calendar (2.3), led discussions between household and community members to support each other's HW (3.2, 3.3), got people to give a public commitment (1.9), and rewarded people who participated and filled in their calendar with a certificate (10.2).  Indirect HWWS Intervention in Schools: Over one week, teachers at local schools talked with students about diarrhea, how it is spread and that it can be preventing via HWWS (4.1, 5.1). The schools infrastructures were improved to include functioning HW stations and buckets for clean and dirty water, and colorful soaps (7.1, 12.1, 12.5). Students formed teams responsible for refilling clean water buckets (3.1, 3.2, 3.3), and were asked to self-monitor their HW with provided calendars (1.4, 2.3). Teachers also monitored students HW (2.1). Students were asked to commit to HW on posters that were displayed in classroom (1.9, 7.1).  Combination: A combination of the above described interventions. | No treatment CG | Staff completed structured observations from 6-9 am for 3 hours. Recorded 1) HW Percent at key time points. 2) HW technique- number of HW steps correctly performed in one HW episode out of 8 steps promoted. 3) Demonstrated HW technique. All measures were recorded pre and post intervention. Analyses reported here only use the post-intervention scores. | Significant effect on HW percentage scores in both direct and combined intervention group.  No significant effect reported for the indirect group. | ★★ |
| 10.Galliani et al. (2016),  RCT [22] | 85  school and community settings  Peru | Total number of children= NA Gender=NA,  Age=NA | One IG experienced a mass media campaign around HW (4.0). Training the trainer activities took place to ensure community based agents of change promoted HW well, i.e. teachers, medical professionals and community leaders. In addition, community-level capacity building through education sessions for mothers, caregivers and children plus HW education in selected primary schools (3.1, 4.0). | Active CG- mass media campaign messages | Enumerators completed structured observations of HWWS over a 4-5 hr period a day after the self-report survey data collected. Measurements of HWWS at key time points prior to eating, prior to food preparation, after fecal contact and before feeding a baby. | Significant difference reported in HWWS before food preparation and before eating vs control condition. | ★★★ |
| 11.Gautam et al. (2017),  RCT [34] | 8  villages  Nepal | 239 mothers,  F= 239,  Age=27 | One IG experienced a food hygiene intervention. The intervention lasted 3 months, and included 2 community events, 4 group meetings, and 6 household meetings with participants focused on the 5 food hygiene behaviors, their consequences, and demonstrations of how to perform them (4.1, 5.1, 6.1). Across the intervention, social support and norms about hygiene were promoted (3.1, 3.2, 3.3, 6.2, 6.3). An overall sense of an 'ideal mother' was created to promote the five hygiene behaviors (13.1) and mothers committed to following them (1.9). The mothers were observed as they practiced the 5 hygiene behaviors and were given feedback on their performance (2.2, 8.1). Additional activities helped mothers learn how to problem solve to avoid anticipated regret from not following the five behaviors (1.2, 5.5) Mothers were provided with prompts and rewards related to the 5 behaviors, e.g. a phone jingle and stickers (7.1, 10.4). Mothers were asked to monitor each other and report what they saw (2.1) and a 'clean kitchen' contest was held with material rewards (10.4, 10.10). At the end of the program, mothers created action plans to follow 5 behaviors and were given feedback on those plans (1.4). | No treatment CG | Observers completed structured observations 1-5 pm. Recorded percent of mothers HWWS before feeding child, percent of children HWWS before eating. For both measures recorded pre and post intervention. | Significant effect on percentage of mothers HWWS and children’s HWWS. | ★★★ |
| 12.Geresomo et al. (2018),  Observational pre-post measurements [35] | 303 Households  Malawi | 40 mothers,  F=40,  Mean Age= 25.3 (SD=7.6) | One IG experienced a food hygiene intervention. In community groups (3.1, 3.2, 3.3) mothers received training on how to prepare food hygienically with locally sourced materials (4.1). The training also emphasized the consequences of unhygienic practices (5.1). Trainers demonstrated how to prepare food hygienically (6.1) and participants received training material kits, e.g. cooking utensils and local food ingredients (12.5). | No CG | Observers completed structured observations of caretakers HWWS – measured pre and post intervention. | Significant effect on HWWS. | ★★★ |
| 13.Graves et al. (2012),  RCT [36] | 51  schools  Kenya | 200 students,  Gender=NA,  Age= Primary school | One IG experienced the NICHE Intervention. During the intervention participants received education on safe water systems, HW and-hygiene more generally (4.0, 4.1). Prompts/cues were provided via posters to promote HWWS in school and at home (7.1) supported by providing the infrastructure and materials for HWWS (12.1). | Delayed intervention CG | Trained NICHE intervention staff members completed structured observation at baseline and 4 months post intervention in intervention and comparison schools. Recorded whether pupils washed their hands after toilet use. | No significant difference reported in IG vs CG for HW after latrine use. | ★ |
| 14.Greenland et al. (2016),  RCT [37] | 16  health centers  Zambia | 306 mothers,  F= 306,  Mean age=28 | One intervention was assessed across 8 cluster groups randomized from 16 health center catchment areas to receive the intervention. The intervention was called the Diarrhea Control Intervention.  Diarrhea Control Intervention: 4 component social marketing campaign:  1. ‘Radio adverts and Call-in shows’ targeting housewives to tell them how to wash their hands and why it is important (4.1, 5.1, 3.1).  2.'Komboni housewives forums' at which women performed skits and talked about their feels toward HW in aim the skits evoke feelings of disgust for not washing hands (3.1, 5.5, 6.1). The activities were supported with small rewards (10.4).  3.'Circle of mothers with monthly prize draw' focused exclusively on breastfeeding with prizes for attendance (10.1, 10.4).  4. 'Roadshows' were similar to the women's forums but included a popular musician and prizes for answering quiz questions correctly (3.1, 3.2, 3.3, 5.5, 6.1, 10.1 10.4). | No treatment CG | Observers complete 3 hr structured observations (one visit post intervention). Recorded HH with soap percent and indicated mothers HWWS after toilet use, or after cleaning up or disposing of a child’s stool. | No significant effect reported for HH with soap percent post intervention. | ★★★ |
| 15.Grover et al. (2018)  RCT [38] | 20  schools  Bangladesh | 3722 children,  F=1883, M=1839,  Age= Primary school | Two out of the four intervention conditions classified as IGs:  Intervention group 1 - restructured the environment to include HW station plus nudge construction sequentially to encourage HW (7.1, 12.1).  Intervention group 2 - received the environmental restructuring of the HW station plus hygiene education sequentially (4.1, 12.1). | 2/4 IGs classified as control conditions | Hidden cameras used to record HWWS after toileting event observed. Measured at 6 points from baseline up to 5 months post intervention. | Significant effect on HWWS for sequential hygiene education and HW station vs simultaneous hygiene education +HW station. | ★★★ |
| 16.Huda et al. (2012),  RCT [39] | 1000 households and community settings  Bangladesh | 1692 caregivers in HH with child <5  Gender=96% F  Age=NA | One IG experienced a community hygiene intervention were HH promoters were trained to deliver messages to main caregivers around water sanitation and-hygiene – (4.0, 4.1) delivered in community settings to encourage social support were caregivers support each other in positive HH behaviors (3.0). | No treatment standard care CG | Research assistant completed structured observations (9-2pm). Recorded HW/HWWS at key time points -after own or child defecation, prior to preparing and serving food and whether wash with or without soap. Measured at baseline and 18 months post intervention. | No significant effect reported on HWWS at key time points. | ★★★ |
| 17.Husain et al. (2018),  RCT [40] | 16  schools  Malaysia | 110 primary school food handlers, F=71; M=8,  Age= NA | One IG experienced a food safety education program focused around the consequences of the food handlers having poor HH (5.0, 5.1). Plus providing information on how to correctly perform HW behaviors (4.1). | No treatment CG | Researchers conducted structured observations to evaluate whether food handlers washed their hands at key time points e.g. before food preparation and if the correct technique was used. Measurements taken at baseline, 6 weeks and 12 weeks. | No significant effect reported for total observed HW practices. | ★★★ |
| 18.Larson et al. (2018),  Observational, pre-post measurements [41] | 3  healthcare facilities  United States of America | 720 pediatric patients,, F=359, M=361, Site 1 mean age = 6; Site 2 mean age = 8; Site 3 mean age = 4 | One IG experienced the Keep it Clean for Kids Intervention. The participants experienced staff team meetings and active participation to support and encourage each other (3.0), education on HH training staff on 5 moments for HH (4.0), visual prompts and cues to action HH (7.1), restructuring of the physical environment to support HH e.g. communication technology that captures HH events mounted into soap (12.1) and hand sanitizer data used to monitor and set goals (1.0, 2.0). | No CG | Communication technology in hand and soap dispensers- used to examine mean HH episodes a day by staff and visitors. Recorded immediately after intervention implementation. | Significant effect on HH episodes recorded post intervention but only maintained significance over time at site 1. | ★★★ |
| 19.Lewis et al. (2018),  RCT [6] | 68  villages  India | 676 mother/child pairs with 1 child age <5 and 1 school age children,  Gender=NA,  Age=NA | One IG experienced a HWWS intervention across a 3 weeks. Students were exposed to education on adventures of 5 super heroes that describe how and when HH should take place (4.1, 4.2). Students were asked to self-monitor HH practice in daily diaries (2.3, 8.1) and their HH monitored at midday meals (2.7) was observed by staff. Students were asked to commit to following the HW steps (1.9) and rewards were provided for the best recordings of HW (10.2). Soap was provided throughout the intervention (12.5) and HH posters displayed where HH should occur (7.2). Female caretakers also asked to attend meetings in week 2 to learn how to HW (4.1), its health-consequences (5.1), and committed to following the HW steps (1.9). | No treatment CG | Female enumerators completed 3hr structured observations in each (5-9am) recording – HH percent: The observers recorded the number of times the school children and their female caretaker experienced the key moments and HW behavior (after defecation, during bathing, and before the three main meals).  Hand-hygiene Percent: Observations carried out in each household at 8–10 weeks post-intervention. | No significant effect reported on HH percentage score. | ★★★★ |
| 20.Linam et al. (2011),  Observational, pre-post measurements  [25] | 2  healthcare facilities (pediatric care units)  United States of America | 1668=unit A  2553=unit B  Healthcare professionals working in pediatric care units  Gender=NA  Age=NA | One IG experienced feedback on their HH on the pediatric care units. The names of non-compliant HH staff were posted (2.0), a team was created to support positive HH compliance (3.0) plus the provision of education and awareness raising of the importance of HH (4.0) plus making sure of availability of HW materials in the units (12.1). | No CG | Covert structured observations of HH compliance defined as acceptable HH before and after contact with patient or patients care environment which included application of alcohol based hand rub product complete coverage of hands and fingers or washing hands with soap without turning off the faucet without using fingers or palms. Patients with contact precaution compliance was defined as gown and gloves donned at room entry then removed and HH performed before exiting room. Performed by patient attendants trained by infection control staff.  Pre and post intervention measurements. | Significant increase in healthcare professionals HH behaviors in unit A (65% to 91% compliance) and unit B (74% to 92% compliance). | ★★ |
| 21.Luby et al. (2018),  RCT [42] | 30  housing compounds  Bangladesh | 692 residents participating with 1 child age<5; 1 school aged child,  F=329, M= 363,  Age=NA | Two IGs called the Soap Intervention and Waterless Hand Sanitizer Intervention. Both interventions initially took place over 9 weeks and then applied again for 5 weeks. Participants were shown findings and pictures of petri-dishes with bacterial growth from contaminated hands (2.2) and then discussed the relationship between HW and child health (5.1). Community members were asked if they wanted to change their behavior (1.9). The next experience depended on the participants groups, as described below.  Soap Intervention: Next, the researchers introduced a bar of soap and explained how and when to use it (4.1). Soap was provided throughout the compounds (12.5) and demonstrated how to use it (6.1). Finally, the mothers from each house were gathered and asked to support each other (3.1). Stickers encouraged HWWS at key locations (7.1), and rewarded households that used most soap with an outside sticker to socially recognize their achievement (10.4, 10.10).  Waterless Hand Sanitizer Intervention: Next, the researchers introduced the alcohol-free waterless hand sanitizer and explained how and when to use it (4.1). Waterless hand sanitizer placed throughout the compounds (12.5) and demonstrated how to use it (6.1). Mothers were gathered and asked to support each other (3.1). Stickers placed at key locations to remind people to sanitize their hands (7.1), outside sticker rewarded the household that used the most sanitizer to socially recognize their achievement (10.4, 10.10). | No treatment CG | Fieldworkers completed covert structured observations (7am-12pm) of overall percentage HH and duration spent HW at key time points - before preparing food, before physical contact with another person, before eating or feeding a child and after cleaning a child’s anus, toileting, sneezing, nose picking, physical contact with another person and returning after at least one hour from outside the compound.  Measurements in each compound on two mornings separated by 2 weeks prior to the intervention, and then post-intervention observations, i.e. follow-up, were conducted twice, 2 weeks apart. | Significant effect reported on soap use for HW and duration of time spent HW increased. | ★★★ |
| 22.Naluonde et al. (2018),  RCT [43] | 50  schools  Zambia | 1101 students,  F=568 M=533,Age=N/A | One IG in which students were given soap on a rope as a hall pass and teachers assessed whether student washed their hands or not (7.1). | Delayed intervention CG | Trained observers conducted structured observations of children’s HW and whether they used soap upon exiting the latrine. Recorded at baseline, time 1 and time 2. | When adjusted for child demographics and water situation no significant difference between IG and CG for HW and HWWS | ★★★★ |
| 23.Oswald et al. (2014),  Observational pre-post measurements  [44] | 27  homes  Peru | 27 mothers,  F= 27  Mean age = 34 | One IG in which participating households received water and sewerage connections (12.1, 12.5). | No CG | Enumerators completed structured observations (over 12 hrs 7-7pm) of HW with OR without soap after defecation and 1 or 15 minutes after fecal hand contamination events and HW with or without soap before handling food after 1 or 15 minutes before handling food. Measured at 3 occasions during both stages of the study (pre and post-intervention). | Significant effect reported for HW after defecation at 1 min but no effect at 15 minutes. | ★ |
| 24.Parvez et al. (2018),  RCT [45] | 432  homes  Bangladesh | 5551 pregnant women,  F=5551,  Age=NA | Six IGs (n=6) that all focused on the following elements, restructuring the physical environment - water treatment and safe storage (12.1), improvement of the sanitation conditions of shared compounds (12.1) and restructuring of the physical environment to provide HW stations (one for the kitchen and one for latrine area plus soapy water bottle and supply of detergent sachets) (12.1). | No treatment CG | Field workers structured observations of HWWS at key time points (5 hour observation period at 15 months post intervention). | Significant effect reported for HWWS. | ★★★ |
| 25.Pickering et al. (2013),  RCT [46] | 6  schools  Kenya | 1364 students,  Gender=NA,  2-13 years old | One IG where a teacher training session about health consequences of HH and germ theory took place (5.1), and appropriate HWWS techniques were demonstrated (6.1) and practiced (8.1). Teachers were provided with culturally sensitive UNICEF HH promotion kits to use in their classrooms to teach students to wash hands with soap (12.5), e.g., posters (7.1), stickers (10.2), a classroom activity book, and a DVD presentation on HW along with a promotional song (4.1, 5.1). Plus soap dispensers were installed and replenished throughout the study (12.1). | No treatment CG | Enumerators completed structured observations (10:30am- 1:30pm 2–4 days a week per school) recording HH percent after toileting and before and after lunch. | Significant effects reported for the percentage of students using sanitizer or soap after the toilet and before lunch and the overall use of sanitizer or soap. | ★★★ |
| 26.Ram et al. (2017),  RCT | 256  homes  Bangladesh | 250 pregnant women,  F=250,  Mean age=20 | One IG experienced behavior change communications based on the health belief model and heuristic model for teachable moments (4.0). In addition the provision of HW stations and soap were made available to support HW (12.1), mothers were told about the importance of HW to encourage a baby to grow healthily (5.0) and were provided with verbal reminders and video cue cards for HW (7.1). | Active intervention CG- maternal and neonatal counselling alone | Structured observations of maternal HWWS and whether one or both hands were washed at times of possible pathogen transmission to the neonate (for 3hrs at 30-32 weeks post-partum). | No significant effect reported for maternal HWWS. | ★★★ |
| 27.Saboori et al. (2013),  RCT [24] | 60  schools  Kenya | 1709 students,  F=872,M=837  Mean age=13 | Two IGs called the HWWS Intervention and the Latrine Cleaning + HWWS Intervention.  HWWS Intervention: Schools received materials to make soapy water (12.5). Teachers were trained how to make soapy water according to a provided training materials, and then taught students how to clean their hands with soap (4.1, 5.1).  Latrine Cleaning + HWWS Intervention: In addition to the HWWS intervention, these participating schools received latrine cleaning supplies (12.1, 12.5) and a binder to self-monitor the latrines cleanliness (2.4). Some teachers were trained to make sure the latrines were clean and encouraged to teach students how to clean them (4.1, 5.1). | No treatment CG | Enumerators conducted structured observations (in 30 minute break period in latrine area) to assess HW/HWWS and scored pupils for HH with soap percent  Observations at baseline and during four monthly visits (aggregated as one follow-up measure in analyses). | Significant effects reported for HH with soap usage for the HW intervention and HW plus latrine intervention. | ★★★ |
| 28.Solehati et al. (2017),  Observational, pre-post measurements [48] | 4  schools  Indonesia | 401 students,  Gender=NA,  Age=Elementary grades 4-6 | A single intervention was implemented across two phases, HWWS Intervention and Training of other students Intervention.  HWWS Intervention: Teachers and a small group of students, called little doctors’ were trained how to wash their hands with soap and then the teachers and little doctors taught other students how to clean their hands with soap (4.1). During training students were able to ask questions (3.1) Video demonstrations (6.1), and posters to prompt HH (7.2) were also made available. *Note that there is not much information given about what training materials were used, beyond the fact that they were co-developed with health workers and local teachers.*  Training of Other Students: The little doctors then trained their peers (6.2). They told students how to clean their hands (4.1) and used the video demonstrations of how to clean one's hands (6.1). Students were also made aware of posters to support and prompt HH (7.2) and were given the chance to ask questions (3.1). | No treatment CG | Structured observations of child HW task pre and post intervention. Recorded percentage with high quality HW: Children asked to perform HW and were assessed using an observation sheet. Assessments were categorized as indicating GOOD or BAD HW. | Significant effect reported for doctor training and student training intervention on percentage of high quality HW behaviors recorded. | ★★★ |
| 29.Watson et al. (2019),  RCT [49] | 2  Sharia compound blocks,  Iraqi Kurdistan | 80 children measured, F=74,M=68,  Age range= 5-12 | One IG experienced an intervention over one weekend. For the intervention, HH promoters delivered 5 transparent soaps with plastic toys hidden inside to households (12.5). During their visit the promoters played the glitter game with children to demonstrate how fast germs transfer (5.1) and informed children about how to wash hands using seven demonstrated steps often (4.1, 6.1). | No treatment CG | Fieldworkers conducted structured observations of randomly selected child from each household (3 hr observation period pre and 4 weeks post intervention). Recorded HH percentage: recorded HW/HWWS at key HH occasions. | Significant effect on percentage of HH episodes. | ★★★ |

^a^Numbers indicate intervention technique given in Additional file 1

*CG= Control group, ED = Environmental-disinfecting, IG= Intervention Group, F= Females, M= Males HH= Hand-hygiene, HW= Handwashing, HWWS= Handwashing with soap
