# Supplementary material for: A systematic review of hand-hygiene and environmental-disinfection interventions in settings with children
Source: BMC Public Health. 2020 Feb 6;20:195. doi: 10.1186/s12889-020-8301-0 (PMC7006391; doi:10.1186/s12889-020-8301-0)
Supplement: Supplementary file 4 — Additional file 4. Quality assessments conducted using the United States Department of Health and Human Services tool for pre-post studies. [file 12889_2020_8301_MOESM4_ESM.docx]

Additional file 4 Quality assessments conducted using the United States Department of Health and Human Services tool for pre-post studies.

| (Pre-Post) studies | Criteria* | | | | | | | | | | | | Stars | |  |
| --- | --- | --- | --- | --- | --- | --- | --- | --- | --- | --- | --- | --- | --- | --- | --- |
|  | 1 | 2 | 3 | 4 | 5 | 6 | 7 | 8 | 9 | 10 | 11 | 12 | |  | |
| Bulled, et al. 2017 [28] |  | ✓ | ✓ | ✓ |  | ✓ |  |  | ✓ | ✓ | ✓ | ✓ | | ★★ | |
| Dreibelbis, et al. 2016 [32] | ✓ | ✓ | ✓ | ✓ |  | ✓ | ✓ |  | ✓ |  |  | ✓ | | ★★ | |
| Geresomo, et al. 2018 [35] | ✓ | ✓ | ✓ |  | ✓ | ✓ | ✓ |  | ✓ | ✓ |  | ✓ | | ★★★ | |
| Larson, et al. 2018 [41] | ✓ | ✓ | ✓ | ✓ |  |  | ✓ | ✓ | ✓ | ✓ | ✓ | ✓ | | ★★★ | |
| Linam, et al. 2011 [25] | ✓ | ✓ | ✓ | ✓ |  |  | ✓ |  | ✓ | ✓ |  | ✓ | | ★★ | |
| Oswald, et al. 2014 [44] | ✓ | ✓ | ✓ |  |  | ✓ |  |  |  |  |  | ✓ | | ★ | |
| Solehati, et al. 2017 [48] | ✓ | ✓ | ✓ | ✓ |  | ✓ | ✓ |  | ✓ | ✓ |  | ✓ | | ★★★ | |
|  |  |  |  |  |  |  |  |  |  |  |  |  | |  | |

*1=Objective clear, 2=Eligibility criteria clear, 3=Sample represents relevant population, 4=All eligible participants enrolled, 5=Sample-size/Power, 6=Consistent implementation of intervention, 7=Outcome assessed multiple times before and after the intervention, 8=Assessors blinded, 9=Loss to follow-up 20% or less, 10=Statistical tests to assess pre-to-post changes, 11=Analysis use individual-level data to determine effects at the group level, 12=Pre-specified and consistent implementation of assessment

*Quality assessments conducted using the United States Department of Health and Human Services tool for controlled interventions*

| Controlled Intervention Studies | Criteria* | | | | | | | | | | | | | | Stars | | |
| --- | --- | --- | --- | --- | --- | --- | --- | --- | --- | --- | --- | --- | --- | --- | --- | --- | --- |
|  | 1 | 2 | 3 | 4 | 5 | 6 | 7 | 8 | 9 | 10 | 11 | 12 | 13 | 14 | |  |  |
| Bieri et al. 2013 [26] | ✓ | ✓ | ✓ |  | ✓ | ✓ |  |  |  | ✓ |  |  | ✓ |  | | ★★★ |  |
| Biran et al. 2014 [27] | ✓ | ✓ | ✓ | ✓ | ✓ |  |  | ✓ |  | ✓ |  |  | ✓ | ✓ | | ★★★ |  |
| Briceño et al. 2017 [23] | ✓ | ✓ | ✓ |  | ✓ |  |  | ✓ | ✓ | ✓ |  | ✓ | ✓ |  | | ★★★ |  |
| Burns et al. 2018 [29] | ✓ | ✓ | ✓ | ✓ |  |  |  | ✓ | ✓ | ✓ |  |  | ✓ |  | | ★★★ |  |
| Caruso et al. 2014 [30] | ✓ | ✓ | ✓ |  | ✓ | ✓ |  |  | ✓ | ✓ |  | ✓ | ✓ |  | | ★★★ |  |
| Chard et al. 2018 [31] | ✓ | ✓ | ✓ | ✓ | ✓ |  |  |  | ✓ | ✓ |  | ✓ | ✓ |  | | ★★★ |  |
| Friedrich et al. 2018 [33] | ✓ | ✓ | ✓ | ✓ | ✓ |  |  |  |  |  |  |  | ✓ |  | | ★★ |  |
| Galliani et al. 2016 [22] | ✓ | ✓ | ✓ | ✓ |  | ✓ |  |  |  | ✓ |  | ✓ | ✓ |  | | ★★★ |  |
| Gautam et al. 2017 [34] | ✓ |  | ✓ | ✓ | ✓ | ✓ |  | ✓ | ✓ | ✓ |  | ✓ | ✓ |  | | ★★★ |  |
| Graves et al. 2012 [36] | ✓ |  | ✓ | ✓ |  | ✓ |  |  |  | ✓ |  |  | ✓ |  | | ★ |  |
| Greenland et al. 2016 [37] | ✓ | ✓ | ✓ |  | ✓ | ✓ |  | ✓ | ✓ | ✓ |  | ✓ | ✓ |  | | ★★★ |  |
| Grover et al. 2018 [38] | ✓ | ✓ | ✓ | ✓ |  | ✓ |  |  | ✓ | ✓ |  | ✓ |  |  | | ★★★ |  |
| Huda et al. 2012 [39] | ✓ | ✓ | ✓ | ✓ | ✓ | ✓ |  |  | ✓ | ✓ |  | ✓ | ✓ |  | | ★★★ |  |
| Husain et al. 2018 [40] | ✓ | ✓ | ✓ | ✓ |  | ✓ |  |  |  | ✓ |  |  | ✓ |  | | ★★★ |  |
| Lewis et al. 2018 [6] | ✓ | ✓ | ✓ | ✓ | ✓ | ✓ |  | ✓ | ✓ | ✓ |  | ✓ | ✓ |  | | ★★★★ |  |
| Luby et al. 2010 [42] | ✓ | ✓ | ✓ | ✓ |  | ✓ |  |  | ✓ | ✓ |  | ✓ | ✓ |  | | ★★★ |  |
| Naluonde et al. 2018 [43] | ✓ | ✓ | ✓ |  | ✓ | ✓ | ✓ | ✓ | ✓ | ✓ |  | ✓ | ✓ | ✓ | | ★★★★ |  |
| Parvez et al. 2018 [45] | ✓ | ✓ | ✓ | ✓ | ✓ | ✓ |  |  | ✓ | ✓ |  | ✓ | ✓ |  | | ★★★ |  |
| Pickering et al. 2013 [46] | ✓ | ✓ | ✓ |  |  | ✓ |  |  | ✓ | ✓ |  | ✓ | ✓ |  | | ★★★ |  |
| Ram et al. 2017 [47] | ✓ | ✓ | ✓ |  | ✓ |  |  |  | ✓ | ✓ |  |  | ✓ |  | | ★★★ |  |
| Saboori et al. 2013 [24] | ✓ | ✓ | ✓ |  | ✓ |  |  |  | ✓ | ✓ |  | ✓ | ✓ |  | | ★★★ |  |
| Watson et al. 2019 [49] | ✓ | ✓ | ✓ | ✓ | ✓ | ✓ |  |  | ✓ |  |  | ✓ | ✓ | ✓ | | ★★★ |  |

*1=Described as randomized, 2=Appropriate method of randomization, 3=Treatment allocation concealed, 4=Group baseline characteristics similar, 5=Sample-size/Power, 6=Consistent implementation of intervention, 7=Participants blinded, 8=Assessors blinded, 9=Loss to follow-up 20% or less, 10=Differential drop-out (between groups) less than 15%, 11=Other interventions avoided, 12=Intention-to-treat analysis, 13=Pre-specified and consistent implementation of assessment, 14=Pre-specified analyses for outcomes and subgroups
